# Clinical efficacy of hysteroscopic adhesiolysis combined with periodic balloon dilation for intrauterine adhesion in IVF treatment

**DOI:** 10.3389/fendo.2023.1236447

**Published:** 2023-09-26

**Authors:** Yuanhui Chen, Yiwen Wang, Yan Zhao, Cuilian Zhang

**Affiliations:** ^1^ Reproductive Medical Center, Henan Provincial People’s Hospital, Zhengzhou, Henan, China; ^2^ Reproductive Medical Center, People’s Hospital of Zhengzhou University, Zhengzhou, Henan, China

**Keywords:** intrauterine adhesions, hysteroscopic adhesiolysis, periodic balloon dilation, embryo transfer, live birth rate

## Abstract

**Background:**

Intrauterine adhesions (IUA), arising from diverse etiological factors, pose a significant threat to female fertility, particularly during *in vitro* fertilization (IVF) treatment.

**Objective:**

To assess the effectiveness of hysteroscopic adhesiolysis (HA) combined with periodic balloon dilation in treating IUA and its impact on reproductive outcomes in women undergoing IVF treatment.

**Methods:**

A total of 234 patients diagnosed with IUA were included in this study. The IUA women were categorized into three subgroups based on the severity of adhesion. All IUA patients underwent HA separation followed by periodic balloon dilation along with hormone replacement therapy (HRT). Frozen embryo transfer was performed post-treatment, and a comparative analysis of the general characteristics and clinical outcomes among the subgroups was conducted. The control group consisted of patients who underwent their first embryo transfer of HRT cycle without any uterine abnormalities, as assessed by the propensity score matching (PSM). The clinical outcomes of IUA group and control group were compared. Multivariate logistic regression analyses were employed to investigate the risk factors associated with live birth.

**Results:**

① The endometrial thickness was significantly increased post-operation compared to pre-operation in all three IUA subgroups (all *P <*0.001), with the most pronounced change observed in the severe IUA group. After treatment, normal uterine cavity was restored in 218 women (93.16%). ② The overall clinical pregnancy rate was 49.57% (116/234) and live birth rate was 29.91% (70/234). The clinical outcomes were similar among the three subgroups after first embryo transfer (all *P*>0.05). Multivariate logistic regression analyses revealed that age (aOR 0.878, 95% CI 0.817~0.944, *P*=0.001) and endometrial thickness after treatment (aOR 1.292, 95% CI 1.046~1.597, *P*=0.018) were the two significant risk factors for live birth rate. ③ Following the process of matching, a total of 114 patients were successfully enrolled in the control group. The baselines and the clinical outcomes were all comparable between the IUA group and control group (all *P*>0.05).

**Conclusion:**

The combination of HA and periodic balloon dilation is beneficial for improving endometrial receptivity and has a significant clinical impact on patients with IUA undergoing IVF.

## Introduction

Intrauterine adhesion (IUA), also known as Asherman’s syndrome, is an endometrial injury disorder that results in the replacement of the underlying endometrial layer with fibrous tissue and the formation of scarring and adhesion bands, leading to deformation and loss of symmetry of the uterine cavity (1). The majority of IUA cases are caused by uterine operations such as abortion and curettage, while a minority result from endoscopic scraping, hysteroscopic surgical operation on the uterus, or uterine artery embolization (2, 3). The condition is characterized by reduced menstrual flow or amenorrhea, infertility, recurrent miscarriage, and spontaneous abortion. These factors have a significant impact on female fertility regardless of whether conception occurs naturally or through assisted reproductive technology.

IUAs leading to amenorrhea following curettage were initially reported by Heinrich Fritsch in 1894 (4). With advancements in technology, HA has become the current gold standard for diagnosing and treating IUA, and has been widely used in clinical practice over recent decades (5). Various methods such as high-dose estrogen replacement therapy, intrauterine devices, water (air) bladders or anti-adhesion agents are frequently employed post-hysteroscopic management to prevent a recurrent adhesion (6).However, despite the implementation of the aforementioned methods, IUA still exhibits a high recurrence rate (ranging from 21.8% to 41.9%), particularly in cases with severe degree of adhesion (7, 8).

Endometrial receptivity is a crucial factor that influences embryo implantation. Women with IUA undergoing fertility treatments present a unique therapeutic challenge and are at high risk for adverse pregnancy outcomes. The negative impact of IUA on reproductive outcomes is primarily due to an avascular and unresponsive endometrium, which exhibits reduced thickness and receptivity, abnormal decidualization, as well as aberrant trophoblastic infiltration. A systematic review including 58 articles has clarified the association between IUA and impaired reproductive outcomes (9). IUAs further aggravate the difficulty of conception for women with fertility, especially for women undergoing IVF treatment with complex etiology. The goal of the surgery is to restore a normal uterine cavity and enhance reproductive outcomes. However, the high probability of re-adhesion following HA separation in IUA significantly impacts the success rate of IVF patients.

In recent years, the Reproductive Medical Center of Henan Provincial People’s Hospital has adopted the technique of HA separation with cold scissor combined with periodic balloon dilation to prevent re-adhesion following adhesion separation, thereby enhancing endometrial receptivity and improving reproductive outcomes. This study aimed to analyze the clinical efficacy of this approach in 234 cases during IVF treatment with different degrees of IUA at our center.

## Materials and methods

### Study design and patients

**Figure 1:**
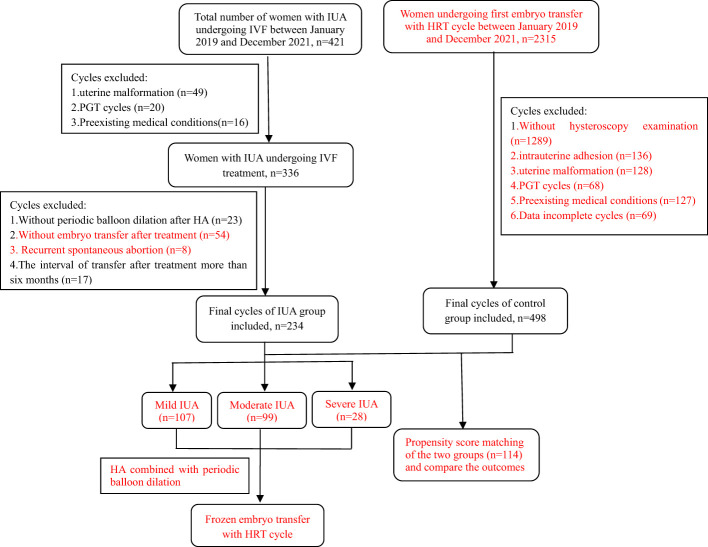
Flow chart of the data selection.

This was an observational study at a single reproductive medical center approved by the Reproductive Medical Ethics Committee of Henan Provincial People’s Hospital. Patients with different degrees of IUA undergoing IVF treatment were enrolled between January 2019 and December 2021. All the IUA patients were further categorized into three subgroups based on the severity of their condition. Diagnostic and classification criteria of IUA was based on Chinese Expert Consensus on Clinical Treatment of Uterine Adhesions in 2015 by the Chinese Society of Obstetrics and Gynecology (10): mild IUA: 0 to 8 points; moderate IUA: 9 to 18 points; severe IUA: 19 to 28 points. The control group consisted of women with normal uterine undergoing their first embryo transfer with artificial cycle, selected through 1:1 propensity score matching (PSM) method. The inclusion and exclusion criteria were shown in detail in **Figure 1**. All couples included in the study signed the relevant informed consent forms.

### Surgical management

The hysteroscopic and balloon dilation surgeries in the study were performed by a skilled surgeon. Hysteroscopic procedures were conducted in an outpatient office setting using cold scissors combined with periodic balloon dilation. Preoperative investigations were conducted, including routine blood tests, leukorrhea analysis, coagulation function assessment, infectious disease screening and electrocardiogram examination to rule out any contraindications for surgery such as reproductive tract inflammation. The surgery was scheduled between 3 to 7 days post-menstruation. In cases of severe IUA accompanied by amenorrhea, artificial cycle therapy was administered prior to the operation in order to stimulate endothelial growth and facilitate intraoperative identification. The surgical equipment utilized in this procedure included the STORZ Continuous Irrigation Single Machine Uterine Cavity Inspection System and Liquid Dilatation Machine Treatment, both of which were manufactured by a reputable German company. Normal saline was used as fluid media, while the dilatation pressure was set at a range of 80~100 mmHg with a flow rate of 200~300 ml/min depending on the uterine size, muscle thickness and stone (11, 12). The uterine cavity morphology, degree of adhesions, endometrial distribution, and bilateral fallopian tube openings were observed and recorded under hysteroscopy. Intraoperatively, patients received HA operation using cold scissors to optimize restoration of the uterine cavity morphology and expose at least one fallopian tube opening.

### Postoperative periodic balloon dilation

Following the procedure, administration of estrogen medication (estradiol valerate 2 mg, twice daily) was initiated, and cyclic balloon dilation was commenced 7 days after HA operation. The dilation involved a double-lumen balloon catheter with a diameter of 14 Fr. To facilitate insertion into the uterine cavity and act as a barrier, approximately 1 cm of the catheter in front of the balloon was removed. The Foley catheter was gently inserted into the uterine cavity and primed with 1 ml of sterile saline as an initial dose. Under ultrasound guidance, additional fluids were incrementally infused into the catheter balloon based on the patient’s tolerance. Fluid administration involved a total volume ranging from 2 to 5 ml, occasionally extending up to 8 ml, followed by a maintenance period of approximately 10 seconds before withdrawal (13). This sequential process was repeated for ten iterations prior to subsequent removal of the balloon catheter. The balloon dilatation was performed once a week on average, for 2 to 3 sessions. For pain-sensitive patients, anti-inflammatory suppositories were inserted rectally prior to surgery to alleviate discomfort. Progestogen was prescribed for seven days following the final balloon dilatation. Subsequently, artificial cycle treatment was initiated during the next menstrual period and a second hysteroscopy examination conducted promptly 3-7 days after the next menstruation.

### Frozen-thaw embryo transfer and luteal support

All patients underwent an artificial regimen to prepare the endometrium for frozen embryo transfer, with a dosage of 4-8 mg/d estradiol valerate tablets initiated on the 2nd to 4th day of menstruation and adjusted according to endometrial thickness (EMT) and morphology. Initiated after a minimum of 11 days to achieve maximum endometrial thickness, vaginal administration of 90 mg/d progesterone gel (Merck Serono, Germany) was supplemented with twice daily oral doses of 10 mg dydrogesterone (Abbott, The Netherlands). Embryo transfer was performed at the optimal timing. Blood β-hCG levels were assessed 14 days post-transfer, and clinical pregnancy defined as the presence of at least one intrauterine gestational sac during weeks 4-5 following transfer. Early miscarriage was defined as loss before 12 weeks gestation. Luteal support medication was discontinued in non-pregnant patients, while continued until 8-10 weeks of pregnancy in those who conceived.

### Statistical analysis

The statistical management and analysis were performed using SPSS software, version 24.0. The measurement data were presented as mean ± standard deviation (mean ± SD). One-way ANOVA or Student’s t-test was used for inter-group comparisons, as appropriate. A paired t-test was employed to assess changes in endometrial thickness before and after treatment. All count data were expressed as percentages (%), and the chi-squared test was utilized to compare count data between groups. The multivariate analysis was conducted using a logistic regression model, and statistical significance was defined as *P* < 0.05. *A* 1:1 PSM was conducted with a matching tolerance of 0.01 for the IUA group and control group. Matching parameters included age and endometrium thickness on transfer day.

## Results

### Study population

The clinical records of patients who met the inclusion criteria were reviewed, and a total of 234 women with IUA undergoing HA combined with periodic balloon dilation treatment and embryo transfer were ultimately enrolled in this study. The IUA patients were classified into three subgroups: the mild group (n=107), the moderate group (n=99), and the severe group (n=28). Additionally, following PSM, a total of 114 patients in the control group without uterine abnormalities after hysteroscopy examination who underwent their first embryo transfer with HRT cycles were enrolled. All participants underwent frozen embryo transfer using artificial cycles. Detailed information regarding the selection process, including the number of cycles and the reasons for inclusion or exclusion, can be found in **Figure 1**.

### Characteristics of IUA patients and efficacy of HA treatment

**Table 1** presented the clinical characteristics of patients with mild, moderate, and severe IUA subgroups. The previous number of uterine surgeries increased significantly with the severity of IUA (*P*=0.040). Following HA operation and periodic balloon dilation, normal uterine cavity was restored in 218 women (93.16%), while 16 women required a second HA procedure (2 in the mild group, 5 in the moderate group, and 9 in the severe group, respectively). As shown in **Table 2**, the endometrial thickness was significantly increased post-operation compared to pre-operation in all three IUA subgroups (all *P <*0.001), with the most pronounced change observed in the severe IUA group (*P*=0.032).

**Table 1:** The clinical characteristics among the IUA groups.

Variables	IUA group			*P value*
	Mild	Moderate	Severe	
No. of cases	107	99	28	
Age (years)	33.1 ± 5.2	34.0 ± 5.1	34.1 ± 6.2	0.492
Duration of infertility (years)	3.0 ± 1.2	3.4 ± 1.0	2.7 ± 0.9	0.414
Basal FSH (IU/L)	6.87 ± 2.85	6.91 ± 2.56	8.14 ± 2.68	0.312
AMH (ng/ml)	3.87 ± 1.97	3.88 ± 1.68	3.16 ± 1.91	0.696
Type of infertility (%)				0.815
Primary infertility	18.7 (20)	22.2 (22)	21.4 (6)	
Secondary infertility	81.3 (87)	77.8 (77)	78.6 (22)	
Gravidity	1.7 ± 1.3	2.0 ± 1.3	2.2 ± 1.6	0.056
No. of uterine surgeries	1.7 ± 1.2	1.8 ± 1.3	2.4 ± 1.5	0.040

**Table 2:** Comparison of endometrial thickness before and after treatment in three IUA groups.

Group	Change of endometrium thickness (mm)	endometrium thickness before treatment (mm)	endometrium thickness after treatment (mm)	*P value*
Mild	0.9 ± 0.7	7.4 ± 1.5	8.4 ± 1.6	<0.001
Moderate	1.2 ± 0.8	6.2 ± 1.7	7.3 ± 1.4	<0.001
Severe	1.6 ± 1.1	4.5 ± 1.3	6.2 ± 1.2	<0.001
*P value*	0.032	<0.001	<0.001	

### Pregnancy outcomes of IUA subgroups

After treatment, all the patients with IUA underwent embryo transfer and their clinical outcomes were presented in **Table 3**. The overall clinical pregnancy rate was 49.57% (116/234) and live birth rate was 29.91% (70/234). The number and stage of embryos transferred, implantation rate, clinical pregnancy rate, early miscarriage rate, late miscarriage rate, ectopic pregnancy rate and live birth rate were comparable across the three groups (all *P*>0.05). Multivariate logistic regression analysis was conducted to identify risk factors associated with live birth rates among the three subgroups. Multivariate logistic regression analysis was conducted to investigate the risk factors associated with live birth rate in three subgroups. The findings revealed that age (aOR 0.878, 95% CI 0.817~0.944, *P*=0.001) and endometrial thickness on transfer day after treatment (aOR 1.292, 95% CI 1.046~1.597, *P*=0.018) were identified as two significant risk factors for live birth rate; however, there was no association between the degree of IUA (*P*=0.823) and live birth following the first embryo transfer (**Table 4**).

**Table 3:** The results of first embryo transfer after treatment in IUA group.

Variables	IUA group			*P value*
	Mild	Moderate	Severe	
No. of cases	107	99	28	
No. of embryos transferred (%)				0.074
1	35.51 (38)	46.46 (46)	57.14 (16)	
2	64.49 (69)	53.53 (53)	42.86 (12)	
Stage of embryos transferred (%)				0.572
Cleavage embryo	47.66 (51)	40.40 (40)	42.86 (12)	
Blastocyst	52.34 (56)	59.60 (59)	57.14 (16	
Implantation rate (%)	42.61 (75/176)	34.87 (53/152)	37.50 (15/40)	0.351
Clinical pregnancy rate (%)	56.07 (60/107)	44.44 (44/99)	42.86 (12/28)	0.187
Early miscarriage rate (%)	28.33 (17/60)	25.00 (11/44)	33.33 (4/12)	0.870^*^
Late miscarriage rate (%)	10.00 (6/60)	6.82 (3/44)	8.33 (1/12)	0.891^*^
Ectopic pregnancy rate (%)	3.33 (2/60)	2.27 (1/44)	8.33 (1/12)	0.766^*^
Live birth rate (%)	32.71 (35/107)	29.29 (29/99)	21.43 (6/28)	0.502

*means Fisher test.

**Table 4:** Multivariate logistic regression analysis of the live birth in IUA group.

	β	Adjusted OR (95% *CI*)	*P value*
Age (years)	-0.130	0.878 (0.817, 0.944)	0.001
Duration of infertility (years)	0.032	1.033 (0.919, 1.161)	0.588
Basal FSH (IU/L)	0.055	1.056 (0.927, 1.205)	0.412
AMH (ng/ml)	0.053	1.054 (0.969, 1.147)	0.218
EMT before treatment (mm)	-0.060	0.942 (0.696, 1.274)	0.697
EMT after treatment (mm)	0.256	1.292 (1.046, 1.597)	0.018
No. of embryo transferred			
1			
2	-0.337	0.714 (0.347, 1.467)	0.359
Stage of embryo transferred			
Cleavage			
Blastocyst	-0.172	0.842 (0.405, 1.748)	0.644
IUA			0.823
1			
2	0.176	1.193 (0.601, 2.365)	0.614
3	-0.089	0.915 (0.281, 2.977)	0.882

### Patients’ clinical baselines and outcomes of IUA and control group after PSM

**Table 5** presented the clinical baseline characteristics and embryo transfer outcomes of both the IUA group and control group after PSM. The basic characteristics were comparable between the two groups including age, duration of infertility, basal follicle-stimulating hormone (FSH), anti-Müllerian hormone (AMH) levels, endometrial thickness, number of embryos transferred and stage of embryos transferred (all *P*>0.05). Also, there were no significant difference in clinical outcomes including implantation rate, clinical pregnancy rate, early miscarriage rate, late miscarriage rate, ectopic rate and live birth rate (all *P*>0.05).

**Table 5:** The comparison of clinical characteristics between IUA group and control group after PSM.

Variables	IUA group	Control group	*P value*
No. of cases	114	114	
Age (years)	33.6 ± 5.5	32.9 ± 5.2	0.286
Duration of infertility (years)	3.2 ± 1.5	3.3 ± 1.7	0.408
Basal FSH (IU/L)	7.04 ± 2.27	6.89 ± 2.05	0.670
AMH (ng/ml)	4.03 ± 1.67	3.79 ± 1.60	0.511
Endometrium thickness (mm)	6.6 ± 0.8	6.7 ± 0.8	0.944
No. of embryos transferred (%)	1.57 ± 0.50	1.53 ± 0.50	0.508
Stage of embryos transferred (%)			
Cleavage embryo	52 (45.61)	58 (50.88)	0.426
Blastocyst	62 (54.39)	56 (49.12)	
Implantation rate (%)	36.31(65/179)	37.35 (62/166)	0.842
Clinical pregnancy rate (%)	45.61 (52/114)	47.37 (54/114)	0.791
Early miscarriage rate (%)	26.92 (14/52)	24.07 (13/54)	0.736
Late miscarriage rate (%)	9.62 (5/52)	7.41 (4/54)	0.739*
Ectopic pregnancy rate (%)	1.92 (1/52)	0 (0/54)	0.491*
Live birth rate (%)	25.44 (29/114)	29.82 (34/114)	0.459

*means Fisher test

## Discussion

The findings of the present investigation suggest that women with IUA undergoing IVF treatment can achieve comparable clinical outcomes after receiving HA treatment combined with periodic balloon dilation, similar to those observed in individuals with comparable baseline. After uterine cavity restoration using HA and subsequent periodic balloon dilation, patients with mild, moderate, or severe IUA demonstrated comparable clinical outcomes after their first embryo transfer cycle with artificial protocol. After adjusting for confounding factors through multivariate logistic regression analysis, both age and endometrium thickness were found to be significantly associated with live birth outcomes.

IUA is a prevalent condition primarily attributed to infectious or traumatic etiologies, exerting significant physical and psychological impacts on women of reproductive age and manifesting symptoms such as hypomenorrhea or infertility. IUA can disturb embryo migration and implantation. Endometrial receptivity plays a critical role in determining successful embryo implantation. The ultrasound-based measurement of endometrial thickness is the most commonly employed method for evaluating endometrial receptivity in clinical settings. The findings of various studies have consistently demonstrated that a thin endometrium is an independent and pivotal risk factor associated with the failure of implantation (14–17). Therefore, the etiology and treatment of thin endometrium have emerged as pivotal and intricate aspects in clinical practice. IUAs are fibrous adhesive bands that develop within the uterine cavity, resulting in the adherence of opposing endometrial surfaces, leading to thinning of the endometrium or other abnormalities which significantly impair endometrial receptivity. The presence of IUAs is strongly associated with a reduced pregnancy rate and an increased risk of pregnancy complications (18). Consequently, diverse strategies have been implemented for the management and restoration of endometrial receptivity in patients with IUAs.

### HA with cold scissor

Currently, HA separation is the first-line standard treatment for IUA. The objective of this procedure is to separate adhesive tissue, restore the anatomical morphology of the uterine cavity and effectively protect the residual endometrium. The separation of HA can be achieved through the utilization of non-electric instruments or electrosurgery, such as monopolar electrotomy, bipolar electrotomy, plasma electrotomy, and micro-scissors. However, there is no consensus on which method is preferable. Non-electric methods involve separating adhesions using non-energy instruments such as micro-scissors, which can avoid the electrothermal effect of energy instruments on normal endometrium around scars and reduce trauma exudation while decreasing postoperative re-adhesion formation. A meta-analysis included nine studies found that hysteroscopic cold scissors was more effective in preventing intrauterine adhesion recurrence, increasing the menstrual flow, reducing intraoperative blood loss, and shortening the operation time than with electrosurgery (19). A randomized trial including 110 patients demonstrated that the thickness of the endometrium in the cold knife group was clearly larger than that in the control group on the 11th and 13th day of menstruation (all *P*<0.05), hysteroscopic cold knife separation can improve the efficacy of IUA and protect the endometrium (20). HA using cold scissors is effective for rebuilding the uterine cavity and provide a fresh and rich blood-supplied surface for endometrium to grow and cover after the surgery (21). In this study, a cold scissor technique was used to minimize the damage caused by energy-based instruments and optimize endometrial protection. This method is characterized by its simplicity, short duration, outpatient setting, high patient compliance, and avoidance of anesthesia risks and serious complications associated with prolonged injection of large volumes of dilatation fluid into the uterine cavity. Therefore, we performed outpatient HA separation using the cold scissor technique.

### Periodic balloon dilation after HA separation

There are various strategies to prevent re-adhesion after IUA separation, including delicate surgical techniques, barrier methods such as gels, intrauterine devices, intrauterine balloons, and stem cell transplantation (6). Foley catheters were initially used for urethral catheterization, but they have recently been widely used in many other clinical applications. The Foley catheter acts as a physical barrier separating the surgical wound and supporting the entire uterine wall to prevent recurrence of adhesion. A randomized controlled trial demonstrated that postoperative intermittent intrauterine balloon dilatation therapy can significantly reduce postoperative adhesion reformation (*P*<0.05) and significantly increase menstruation flow (*P*<0.001) (22). Moreover, the placement of pediatric Foley catheter after HA does not result in ascending infection (23).

In our center, we utilize a cold scissor separation technique in conjunction with periodic balloon dilation treatment, which is non-thermal throughout the entire process. Periodic balloon dilation is performed one week post-surgery for 2-3 times every month to act as a barrier and dilate the uterine cavity, thereby preventing adhesion formation during the short period after separation and enhancing endometrial blood flow. In this study, we observed that all three IUA subgroups exhibited significant improvement in endometrial thickness after HA separation combined with periodic balloon dilation. Moreover, the recovery rate of uterine cavity morphology surpassed 90% in both mild and moderate IUA groups, which was significantly superior to that of the severe IUA group. Therefore, our findings suggest a more practical clinical treatment approach for women suffering from mild to moderate IUA.

### Embryo transfer of HA treatment

Endometrial receptivity is a crucial factor influencing the outcomes of IVF, and research has shown that endometrial thickness plays a significant role in determining the success rates of embryo transfer following HA treatment (24). A study involving 357 patients with different degrees of IUA revealed an overall pregnancy rate of 48.2% following HA treatment, which gradually declined as the severity of IUA increased (25). For individuals who have undergone IVF following HA separation, several studies indicated that the clinical pregnancy rate after embryo transfer may be approximately 50% (26, 27), which is a relatively satisfactory outcome in clinical practice.

A total of 234 infertile women with IUA were enrolled in this study. The clinical outcomes showed no significant differences among different degrees of IUA groups after treatment with HA separation combined with periodic balloon dilation, which differs from the findings reported in previous studies (28), maybe related to the sample size and needs to be further investigated, or some patients without embryo transfer were excluded.

The further adjusted analysis revealed that age and endometrial thickness were two significant risk factors for live birth rate after embryo transfer, regardless of the degree of intrauterine adhesion (IUA). Wang’s study also demonstrated that age and EMT had a considerable impact on pregnancy outcomes (24). Insufficient endometrial receptivity is the primary factor affecting embryo implantation (29). The restoration of uterine cavity, as well as increasing EMT and endometrial blood flow, are crucial steps to improve implantation success. To evaluate the clinical outcomes after treatment, we selected the control group through PSM method, matching age and endometrium thickness, and the results showed that implantation rate, clinical pregnancy rate, miscarriage rate, and live birth rate were similar between IUA group and control group. Therefore, the outcome of embryo transfer following treatment may be comparable to that of individuals with similar baselines but without uterine abnormalities. It can be speculated that HA combined with periodic balloon dilation treatment maybe an effective approach for IUA patients.

Some limitations existed in our study. Firstly, the study was not originally designed as a randomized controlled trial, and thus the control group may not be considered optimal. Ideally, the control group should consist of women with untreated IUA who undergo embryo transfer, allowing for a more comprehensive evaluation of the treatment’s effectiveness in this study. However, conducting embryo transfer without any intervention for women with IUA contradicts medical principles and ethical considerations. Consequently, we employed PSM to establish comparability between the control group and IUA group, thereby facilitating an assessment of the clinical outcomes after embryo transfer. Moreover, the sample size was small, and subsequent large-scale studies are imperative to further validate the findings.

## Conclusions

The combination of cold instrumentation and periodic balloon dilation proves to be an effective approach in restoring the uterine cavity and enhancing endometrial receptivity for women undergoing IVF combined with IUA. After treatment, satisfactory clinical outcomes can be achieved following embryo transfer, particularly for patients with mild uterine adhesions. This method holds significant potential for clinical promotion.

## Data availability statement

The original contributions presented in the study are included in the article/supplementary material. Further inquiries can be directed to the corresponding author.

## Ethics statement

The studies involving humans were approved by Reproductive Medical Ethics Committee of Henan Provincial People’s Hospital. The studies were conducted in accordance with the local legislation and institutional requirements. Written informed consent for participation was not required from the participants or the participants’ legal guardians/next of kin in accordance with the national legislation and institutional requirements.

## Author contributions

YC designed the study and wrote the manuscript. YW and YZ were involved in the data collection and analysis. CZ was responsible for providing data and guiding research. All authors contributed to the article and approved the submitted version.

## References

[1] Ma J Zhan H Wen L Zhang L Yun F Wu R. Recent trends in therapeutic strategies for repairing endometrial tissue in intrauterine adhesion. Biomater Res (2021) 25(1):40. doi: 10.1186/s40824-021-00242-6 34819167 PMC8611984

[2] Deans R Abbott J. Review of intrauterine adhesions. J Minimally Invasive Gynecol (2010) 17(5):555–69. doi: 10.1016/j.jmig.2010.04.016 20656564

[3] Rein DT Schmidt T Hess AP Volkmer A Schöndorf T Breidenbach M. Hysteroscopic management of residual trophoblastic tissue is superior to ultrasound-guided curettage. J Minim Invasive Gynecol (2011) 18(6):774–8. doi: 10.1016/j.jmig.2011.08.003 22024264

[4] Fritsch H. Ein Fall von volligen Schwund der Gebaumutterhohle nach Auskratzung. Zentralbl Gynaekol (1894) 18:1337–42.

[5] AAGL Elevating Gynecologic Surgery. AAGL practice report: practice guidelines on intrauterine adhesions developed in collaboration with the european society of gynecological endoscopy (ESGE). J Minim Invasive Gynecol (2017) 24(5):695–705. doi: 10.1016/j.jmig.2016.11.008 28473177

[6] Lee WL Liu CH Cheng M Chang WH Liu WM Wang PH. Focus on the primary prevention of intrauterine adhesions: current concept and vision. Int J Mol Sci (2021) 22(10):5175. doi: 10.3390/ijms22105175 34068335 PMC8153321

[7] Hanstede MMF Meij E Goedemans L Emanuel MH. Results of centralized Asherman surgery, 2003-2013. Fertil Steril (2015) 104(6):1561–8.e1. doi: 10.1016/j.fertnstert.2015.08.039 26428306

[8] Zhou Q Shi X Saravelos S Huang X Zhao Y Huang R. Auto-cross-linked hyaluronic acid gel for prevention of intrauterine adhesions after hysteroscopic adhesiolysis: A randomized controlled trial. J Minim Invasive Gynecol (2021) 28(2):307–13. doi: 10.1016/j.jmig.2020.06.030 32681996

[9] Hooker AB Leeuw RA Emanuel MH Mijatovic V Brolmann HAM Huirne JAF. The link between intrauterine adhesions and impaired reproductive performance: a systematic review of the literature. BMC Pregnancy Childbirth (2022) 22(1):837. doi: 10.1186/s12884-022-05164-2 36376829 PMC9664654

[10] Chinese Society of Obstetrics and Gynecology Chinese Medical Association. Expert consensus on the diagnosis and management of intrauterine adhesions in China. Zhonghua Fu Chan Ke Za Zhi (2015) 50(12):881–7. doi: 10.3760/cma.j.issn.0529-567x.2015.12.001 26887869

[11] Umranikar S Clark TJ Saridogan E Miligkos D Arambage K Torbe E. BSGE/ESGE guideline on management of fluid distension media in operative hysteroscopy. Gynecol Surg (2016) 13(4):289–303. doi: 10.1007/s10397-016-0983-z 28003797 PMC5133285

[12] AAGL Advancing Minimally Invasive Gynecology Worldwide Munro MG Storz K Abbott MG Falcone T Jacobs VR. AAGL practice report: practice guidelines for the management of hysteroscopic distending media: (Replaces hysteroscopic fluid monitoring guidelines. J Am Assoc Gynecol Laparosc (2000) 7:167–8. doi: 10.1016/j.jmig.2012.12.002 23465255

[13] Ludwin A Martins WP Ludwin I. Ultrasound-guided repeat intrauterine balloon dilatation for prevention of adhesions. Ultrasound Obstet Gynecol (2019) 54(4):566–8. doi: 10.1002/uog.20223 30677188

[14] Ebovitz OL Orvieto R. Treating patients with “thin” endometrium - an ongoing challenge. Gynecol Endocrinol (2014) 30(6):409–14. doi: 10.3109/09513590.2014.906571 24693854

[15] Vartanyan E Tsaturova K Devyatova E. Thin endometrium problem in IVF programs. Gynecol Endocrinol (2020) 36(sup1):24–7. doi: 10.1080/09513590.2020.1816724 33305667

[16] Kasius A Smit JG Torrance HL Eijkemans MJC Mol BW Opmee BC. Endometrial thickness and pregnancy rates after IVF: a systematic review and meta-analysis. Hum Reprod Update (2014) 20(4):530–41. doi: 10.1093/humupd/dmu011 24664156

[17] Liu KE Hartman M Hartman A Luo Z Mahutte N. The impact of a thin endometrial lining on fresh and frozen-thaw IVF outcomes: an analysis of over 40 000 embryo transfers. Hum Reprod (2018) 33(10):1883–8. doi: 10.1093/humrep/dey281 PMC6145412 30239738

[18] Evans-Hoeker EA Young SL. Endometrial receptivity and intrauterine adhesive disease. Semin Reprod Med (2014) 32(5):392–401. doi: 10.1055/s-0034-1376358 24959821

[19] Yang L Wang L Chen Y Guo X Miao C Zha Y. Cold scissors versus electrosurgery for hysteroscopic adhesiolysis: A meta-analysis. Med (Baltimore) (2021) 100(17):e25676. doi: 10.1097/MD.0000000000025676 PMC8084071 33907137

[20] Jin X Ye JL Zhang L Chen LC. Efficacy of hysteroscopic cold knife separation on intrauterine adhesions. Am J Transl Res (2021) 13(7):8351–7. PMC8340206 34377327

[21] Zhang A Jamail G Xue M Guan XM Xiao SH Xu DB. Hysteroscopic intrauterine adhesiolysis using the “Ploughing” Technique with cold scissors. J Minim Invasive Gyneco (2015) 22(6):934–5. doi: 10.1016/j.jmig.2015.05.009 25999022

[22] Shi X Saravelos SH Zhou Q Huang X Xia E Li TC. Prevention of postoperative adhesion reformation by intermittent intrauterine balloon therapy: a randomized controlled trial. BJOG (2019) 126(10):1259–66. doi: 10.1111/1471-0528.15843 31207009

[23] Abuzeid OM Hebert J Ashraf M Mitwally M Diamond MP Abuzeid M. Pediatric foley catheter placement after operative hysteroscopy does not cause ascending infection. J Minim Invasive Gynecol (2018) 25(1):133–8. doi: 10.1016/j.jmig.2017.06.031 28847756

[24] Wang XC Yi JS Xie X Du S Li L Zheng X. Factors affecting pregnancy outcomes following the surgical removal of intrauterine adhesions and subsequent in *vitro* fertilization and embryo transfer. Exp Ther Med (2019) 18(5):3675–80. doi: 10.3892/etm.2019.7935 PMC6777258 31602246

[25] Chen L Zhang HW Wang Q Xie F Gao S Song Y. Reproductive outcomes in patients with intrauterine adhesions following hysteroscopic adhesiolysis: experience from the largest Women’s Hospital in China. J Minim Invasive Gynecol (2017) 24(2):299–304. doi: 10.1016/j.jmig.2016.10.018 27856386

[26] Fouks Y Kidron A Lavie I Shapira Z Cohen Y Levin I. Reproductive outcomes and overall prognosis of women with asherman’s syndrome undergoing IVF. J Minim Invasive Gynecol (2022) 29(11):1253–9. doi: 10.1016/j.jmig.2022.08.004 35970266

[27] Wang Y Yao Z Zhao H Yue C Yu Q Zhang Y. Reproductive outcomes of *in vitro* fertilization-intracytoplasmic sperm injection after transcervical resection of adhesions: A retrospective cohort study. J Minim Invasive Gynecol (2021) 28(7):1367–74. doi: 10.1016/j.jmig.2020.10.029 33188921

[28] Hanstede MMF Meij E Veersema S Emanuel MH. Live births after Asherman syndrome treatment. Fertil Steril (2021) 116(4):1181–7. doi: 10.1016/j.fertnstert.2021.05.099 34130799

[29] Guardo FD Palumbo M. Asherman syndrome and insufficient endometrial thickness: A hypothesis of integrated approach to restore the endometrium. Med Hypotheses (2020) 134:109521. doi: 10.1016/j.mehy.2019.109521 31887722

